# Eccrine Poroma of the Palm: A Case Report and Literature Review

**DOI:** 10.7759/cureus.69489

**Published:** 2024-09-15

**Authors:** Neeraj Mishra, Darryl Ee Ming Chew, Kenneth Pak Leung Wong, Mohammad Ashik Bin Zainuddin

**Affiliations:** 1 Orthopaedic Surgery, Kandang Kerbau (KK) Women's and Children's Hospital, Singapore, SGP; 2 Hand and Reconstructive Microsurgery, Singapore General Hospital, Singapore, SGP

**Keywords:** benign tumor, eccrine poroma, hand, hand tumor, palm, poroma

## Abstract

Eccrine poroma is a non-cancerous tumor that arises from the intraepidermal portion of the eccrine sweat glands. It usually appears as a solitary lesion on an extremity, commonly on the foot or sole, and is often subject to delayed or inaccurate diagnosis in clinical settings. This article describes a rare case of eccrine poroma located on the palm. It discusses the clinical and histological features, diagnostic difficulties, recurrence risks, and the possibility of malignant transformation associated with this condition.

## Introduction

Eccrine poroma is a non-cancerous tumor originating from the intraepidermal portion of the eccrine sweat glands, which is also known as the acrosyringium [1]. This tumor typically presents as a solitary nodule that gradually increases in size and may become ulcerated over time [1]. It most frequently occurs on the extremities, with a notable tendency to appear on the foot or sole [2,3]. This slow-growing, often ulcerated lesion can make early diagnosis challenging, highlighting the need for careful clinical evaluation and consideration of eccrine poroma in the differential diagnosis of similar skin lesions. Eccrine poroma is often dubbed the "great imitator" due to its ability to closely resemble various benign and malignant skin tumors, both in clinical appearance and dermoscopic patterns. This resemblance frequently complicates the diagnostic process, making histopathological evaluation essential to differentiate it from other skin lesions accurately. In this case study, we present a rare instance of eccrine poroma located on the palm of a middle-aged man, as observed, and treated at our hospital. This unusual presentation underscores the importance of considering eccrine poroma in the differential diagnosis of palm lesions and highlights the challenges in achieving a precise diagnosis.

## Case presentation

A 54-year-old, right-handed Chinese man, with no notable medical history, sought medical attention for a painless mass on his left palm. This swelling had been progressively enlarging over the past two years. The patient reported that the lump had not exhibited any symptoms such as discharge, bleeding, crusting, ulcers, or itching, and there had been no alterations in its shape or color. He also recalled a prior incident where his palm was punctured by a fishbone before the onset of this swelling.

**Figure 1:**
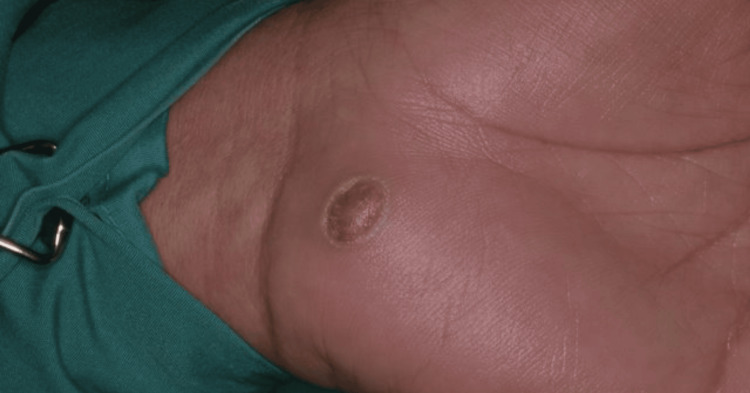
Benign eccrine poroma of the left palm

Upon clinical evaluation, a distinct, non-tender, firm, oval-shaped papule was identified in the hypothenar region of the left palm, measuring 18 mm at its widest point (Figure 1). The lesion exhibited mild erythema and was accompanied by adjacent hyperkeratosis. There were no symptoms or signs pointing to infection or malignancy. Both motor and sensory functions of the hand were intact, and no lymphadenopathy was present. Initially, a provisional diagnosis of a pyogenic granuloma was considered, with implantation dermoid cysts as a differential diagnosis.

The patient underwent an excisional biopsy of the mass, conducted under wrist block anesthesia to reduce the risk of cancer dissemination if a malignant diagnosis was confirmed. The procedure involved the complete removal of the mass along with a 3 mm margin of adjacent healthy tissue, using an elliptical incision. The incision site was subsequently closed with primary sutures.

Histological examination revealed a benign adnexal tumor characterized by a unique pattern of cuticular and eccrine glandular cells, which organized into duct-like formations containing secretions within their lumens. The tumor exhibited well-defined margins.

Post-surgery, the patient experienced a smooth recovery and was closely monitored over a two-year period. During follow-up visits, he continued to be in excellent health with no indications of tumor recurrence.

## Discussion

Pinkus et al. first reported benign eccrine poroma in 1956 [1]. There has been some debate regarding the preferred locations of these tumors in the body. The prevailing view suggests that eccrine poromas primarily occur on smooth, hairless acral areas like the hands and feet, attributed to the high density of eccrine sweat glands in these regions [2]. Nonetheless, various case series have shown that the head, neck, and trunk are more frequent sites for eccrine poromas than previously thought [3].

According to the cases documented, there seems to be no significant association between these lesions and factors such as sex, race, or family history. Additionally, eccrine poromas have been observed across all age groups, though they most commonly occur in individuals in their seventies [3].

Clinically, larger eccrine poromas and those located in atypical areas are quite rare and challenging to diagnose without histological examination. While giant eccrine poromas do exist, they are exceedingly uncommon [4]. Another infrequent variant is eccrine poromatosis, characterized by the presence of numerous (over 100) papules distributed across the body [5,6]. This condition often manifests in patients who have received chemotherapy or radiotherapy. Although the precise cause remains uncertain, it is hypothesized that the frequent development of eccrine poromas might be associated with the prolonged effects of these treatments, rather than being strictly a paraneoplastic phenomenon. Some studies suggest that toxic metabolites from chemotherapy, which accumulate in eccrine glands, could lead to cellular changes and the eventual formation of tumors [7].

Diagnosing eccrine poromas clinically can be challenging due to their varied appearances, which can resemble a wide range of other skin conditions. These lesions may resemble pyogenic granulomas, dermoid cysts, acrochordon (skin tags), verruca vulgaris (warts), seborrheic keratosis, fibromas, melanomas, adnexal cysts, vascular tumors, basal cell carcinomas, and squamous cell carcinomas. Typically, eccrine poromas present as skin-colored papules or nodules measuring less than 2 cm in diameter. However, they can also appear pigmented due to melanin or erythematous from blood vessel dilation or proliferation [8,9]. In some cases, these lesions may exhibit surface erosion or ulceration, which is often attributed to trauma.

From a histological perspective, eccrine poromas are characterized by their structure of extensive, interconnecting bands of evenly sized, cuboidal epithelial cells. These cells feature round nuclei that are lightly basophilic and are surrounded by a moderate amount of cytoplasm that ranges from pale to eosinophilic in color. Often, the tissue shows areas where the cells display early stages of tubular differentiation, indicating the formation of small tubular structures within the tumor.

The treatment approach for eccrine poromas involves thoroughly removing the tumor along with a narrow margin of healthy skin and underlying tissue to ensure complete excision. Recurrence following inadequate excision has occurred in various parts of the body [10-12]. Therefore, it is crucial for patients to undergo regular follow-up visits to monitor for any signs of recurrence or the emergence of new lesions elsewhere in the body.

If these lesions exhibit symptoms such as pain, bleeding, ulceration, itching, or rapid growth over a short period, the possibility of malignant transformation, such as eccrine porocarcinoma or malignant eccrine poroma, should be considered [13]. Literature reports have documented cases where histologically confirmed transformations from benign to malignant tumors have occurred in other parts of the body [14]. Orella et al. propose that given the often lengthy period before diagnosis, these lesions may have developed from previously benign eccrine poromas [15]. Eccrine porocarcinoma is most commonly observed in the lower extremities (44%), trunk (24%), and head (18%). Fewer cases have been noted in the upper extremities (8%) and hands (3%) [16].

Clinically, eccrine porocarcinoma is typically observed as warty plaques or pedunculated growths that can bleed easily from minor injuries [17]. These malignant lesions may initially present in an in-situ stage before progressing. Eccrine porocarcinomas are extremely rare compared to their benign counterparts and arise from the intraepidermal ductal segment of the eccrine sweat gland. The incidence of eccrine porocarcinoma is remarkably low, accounting for less than 0.01% of all skin biopsy samples [18,19]. The condition is associated with a 50% likelihood of metastasis to regional lymph nodes, and the overall prognosis tends to be unfavorable [20]. Additionally, porocarcinoma often has a high recurrence rate even after surgical intervention. Treatment strategies include standard surgical excision, wide local resection, Mohs micrographic surgery, radiation therapy, and, in extreme cases, amputation.

## Conclusions

Benign eccrine poromas affecting the hand are relatively rare. Our review of medical literature from 1956 to 2022 identified only nine documented cases of eccrine poromas on the palm. None of these cases had any history of prior trauma. In contrast, our patient reported a history of being pricked by a fishbone before the development of the lump. Given the infrequent occurrence of this lesion on the hand, surgeons need to recognize its atypical presentation and be vigilant for signs of potential malignant transformation. Eccrine poroma should be considered in the differential diagnosis of chronic hand lesions, even if there is a history of trauma. Ensuring complete excision is essential to prevent recurrence, address any dysplastic changes, and reduce the risk of future malignant transformation.
